# Water, Sanitation, and Hygiene Service Availability at Rural Health Care Facilities in Southwestern Uganda

**DOI:** 10.1155/2018/5403795

**Published:** 2018-08-27

**Authors:** Edgar Mugema Mulogo, Micheal Matte, Andrew Wesuta, Fred Bagenda, Richard Apecu, Moses Ntaro

**Affiliations:** ^1^Department of Community Health, Mbarara University of Science and Technology, P.O. Box 1410, Mbarara, Uganda; ^2^Department of Medical Laboratory Science, Mbarara University of Science and Technology, P.O. Box 1410, Mbarara, Uganda

## Abstract

There is a paucity of information on the state of water, sanitation, and hygiene (WASH) at health care facilities in Uganda. A survey on WASH service availability was conducted at 50 health care facilities across 4 districts of rural southwestern Uganda between September and November 2015. The main water points at the majority (94%) of the health care facilities were improved sources, while improved toilets were available at 96% of the health care facilities visited. Hospitals had the poorest toilet to patient ratio (1 : 63). Only 38% of the health care facilities had hand washing facilities at the toilets. The lack of hand washing facilities was most prominent at the level IV health centre toilets (71%). Hand washing facilities were available at other points within most (76%) of the health care facilities. However, both water and soap were present at only 24% of these health care facilities. The poor toilet to patient/caregiver ratios particularly in the high volume health care facilities calls for the provision of cheaper options for improved sanitation in these settings. Priority should also be given to the sustainable provision of hygiene amenities such as soap for hand washing particularly the high patient volume health care facilities, in this case the level IV health centres and hospitals.

## 1. Background

WASH services provide for water availability and quality, presence of sanitation facilities, and availability of soap and water for hand washing [1]. A joint WHO/UNICEF report shows that globally, provision of WASH services in health care facilities is low, and the current levels of service are far less than the required 100% coverage by 2030. The report also notes that large disparities in WASH services in health care facilities exist between and within countries [2]. Provision of water is lowest in the African Region, with 42% of all health care facilities lacking an improved source on-site or nearby. However, provision of sanitation services was much better with only 16% of all health care facilities in the African Region lacking access to improved sanitation [2]. It has also been reported that large variations have been observed at subnational level, by settings and by type of health care facility within the same country, with smaller facilities in rural areas having disproportionally fewer WASH services compared to larger facilities (e.g., hospitals) in urban areas [2].

Almost half the people in the developing world have one or more of the main diseases or infections associated with inadequate water supply and sanitation [3]. Inadequate drinking water, sanitation, and hygiene in nonhousehold settings, such as schools, health care facilities, and workplaces, impact the health, education, welfare, and productivity of populations, particularly in low- and middle-income countries [4, 5].

Health-care-associated infections affect hundreds of millions of patients every year, with 15% of patients estimated to develop one or more infections during a hospital stay [6]. Unsafe water and sanitation and poor hygiene practices in health care facilities lead to health-care-acquired infections [7]. Previous studies also show that compliance with hand washing standards among health care providers is often low and health care providers often transmit infection. As a result, health care facilities are a source of infection and patients seeking treatment fall ill, and potentially die, for the lack of basic elements of a safe and clean environment [5]. Further, poor water and sanitation infrastructures and hygiene practices at health care facilities affect health care seeking behavior among catchment communities due fear of contracting infections [8].

Globally, improving water, sanitation, and hygiene has the potential to prevent at least 9.1% of the disease burden (in disability-adjusted life years or DALYs), or 6.3% of all deaths [9]. A report by the WHO suggests that higher levels of WASH services can significantly reduce diarrheal illness [10]. The availability of WASH services at health care facilities is essential for preventing and treating disease and therefore directly reduces the disease burden [5, 11–13].

The responsibility for delivery of WASH interventions in Uganda is shared between the Ministries of Education (WASH in schools), Health (community sanitation), and Water and Environment (infrastructure and public sanitation plus sewerage services). A memorandum of understanding (MOU) that was signed by the three ministries in 2001 on sanitation has not translated into an improved sanitation situation as there have been challenges in coordination and resource commitments [14]. Uganda's Local Government Act (1997), as amended, mandates local governments at district and subcounty levels to provide services including WASH to the community and the institutions and to provide adequate support for operation and maintenance of water systems by the users in liaison with the Ministry of Water and Environment [14].

In the past, measuring access to WASH has mainly focused on the household level [15], and therefore WASH coverage statistics for Uganda generally highlight household data. The data available for coverage for WASH at health facilities in Uganda were previously derived from a Service Provision Assessment conducted in 2007 [2]. A multicountry study has recently provided statistics on WASH in health care facilities in Uganda [7]. However, there continues to be a paucity of information on status of WASH at health care facilities in Uganda. This study evaluates availability of WASH services at government-owned health facilities in a rural context, almost a decade after the first assessments were made in 2007. The JMP Post-2015 Working Group proposes that by 2030 all health centres provide all users with basic drinking water supply, adequate sanitation facilities, and hand washing and menstrual hygiene facilities [16]. The WHO suggests that it is important to first understand the extent of the problem and subsequently prioritize action where needs are greatest [2]. The study findings will therefore contribute to decision-making processes for the implementation of WASH standards at health facilities in Uganda.

## 2. Materials and Methods

### 2.1. Design and Health Care Facility Selection

A cross sectional survey was conducted across 50 health facilities in 4 districts of southwestern Uganda. The districts include Ibanda, Isingiro, Kiruhura, and Mbarara. The districts have a total area of 10,019.1 sq. km in size and a population of 1,460,100 of which 51% are female [17]. The population under five and females aged 15–44 years constitute 15.3% and 25% of the population, respectively. The majority of the population (82–89%) resides in rural areas. The wet season in this area usually occurs from March to May and September to November with the dry seasons in between these periods. Access to safe water varies among the districts as follows: Isingiro 28%, Kiruhura 31%, Ibanda 57%, and Mbarara 70%. The main water supply technologies are public stand posts, protected spring technology, and deep boreholes. Rain harvesting tanks, gravity flow schemes, and in a few cases groundwater-based pumped piped water supply system are present [18]. Latrine coverage among these districts varies from 75 to 93% of households [17].

The health service infrastructure follows the pattern of the administrative units, with health centres (HCs) of increasing capacity (designated level II HC, level III HC, and level IV HC), general hospitals, and a regional referral hospital. The catchment populations for the different levels of health facility vary from 5000 for the level II HC to 500,000 for the general hospitals [19].

The level III HCs (catchment population 20,000) provide basic preventive, promotive, and curative care. They also provide support supervision of the community and the level II HCs under their jurisdiction. There are provisions for laboratory services for diagnosis, maternity care, and first referral cover for the subcounty [19]. The level III HCs have limited inpatient capacity mainly maternity and general patient wards. The level II HCs (catchment population 5,000) are stand-alone facilities that provide the first level of interaction between the formal health sector and the communities. The level II HCs only provide outpatient care and community outreach services [19]. The level I HCs comprise village health teams (VHTs) and have no physical infrastructure. The VHTs facilitate health promotion, service delivery, community participation, and empowerment in access to and utilization of health services [19].

The level IV HCs (catchment population 100,000) provide outpatient and inpatient services, have maternity, adult, and children's wards and an operating theatre, and provide laboratory and blood transfusion services [19, 20]. The general hospitals (catchment population 500,000) provide preventive, promotive, curative, maternity, and inpatient health services and surgery, blood transfusion, laboratory, and medical imaging services. They also provide in-service training, consultation, and operational research in support of the community-based health care programmes [19].

There are a total of 282 health care facilities in the 4 study districts. The majority of the facilities (88%) are government owned and the rest privately owned [21]. The 50 randomly selected health care facilities comprised the level III and level IV HCs and a general hospital. The selection of the number of the different facilities was related to patient volume, and more of the lower volume facilities (level III) were selected. All the health care facilities selected were government owned. The facilities were visited between September and November 2015.

### 2.2. Instruments, Variables, and Analysis

To assess availability of WASH services in each facility, a standardized observation checklist dealing with availability, technology, and condition was filled out during a visit to all forms of water supply and sanitation used by the health care facilities. The managers of the health care facilities were interviewed in order to assess reliability of the water supply and other factors affecting the state and use of the WASH services, such as duration WASH facilities had been in place and presence of WASH committees. Record reviews were undertaken to collect supplementary information on health facility patient load.

The availability of WASH services was assessed based on an adaption of indicators used in a report by the WHO on WASH in health care facilities [2]. The services were defined as follows:

- *Water*. The presence of an improved water source or water supply within the facility (in building or compound) used for drinking, personal hygiene, medical activities, cleaning, laundry, and cooking. The functionality (water was available from this source at the time of the survey), mean distance to sources from inpatient ward, and mean queuing time were also assessed, and alternative options for water storage and the availability of water point maintenance plans were also assessed. The assessment of all these indicators was made through observation.
- *Sanitation*. The presence of latrines or toilets within the facility, distance from outpatient departments and inpatient wards, toilet to patient ratio, cleanliness, availability of cleaning materials, availability of separate toilets for males, females, and disabled, capability to close and lock, availability of lighting at night, and extent of filling for pit latrines through observation. The mechanism of emptying the toilets was established through an interview with the facility managers.
- *Hygiene*. The availability of hand washing facilities with soap or alcohol-based rubs at the toilets and within the facility buildings was assessed through observation.

The analysis was run using Stata version 12 version [22]. To compare the means of variables such as health facility patient numbers and number of water points and toilet stances available, *t*-tests were conducted. The means were computed in order to evaluate service availability in terms of patients per water point or patients per latrine stance. This enabled comparisons with recommended WHO standards [12]. Water and sanitation service availability was categorized as improved and unimproved, and findings were reported on selected indicators for monitoring WASH in health care facilities [23]. These included whether the main point of water was an improved source, water point was located on premises, there were improved toilets, toilets were located on premises, and hand hygiene stations were present and located within 5 meters of toilet.

While two-sided chi-square tests for association were computed to evaluate the association between the presence of WASH risk assessment and maintenance plans and WASH service availability, capability to close toilets and the cleanliness of toilets, availability of toilet lighting and cleanliness of toilets, among others. Significance was set at a probability value (*p* value) level of 0.05.

#### 2.2.1. Ethics Approval and Consent to Participate

Ethical approval for this study was sought and obtained from the Research Ethics Committee at Mbarara University of Science and Technology. Written informed consent was also obtained from the individual subjects.

## 3. Results

### 3.1. Health Facilities Visited

The majority (62%) of facilities visited were level III HCs. The types of facility visited and the mean and median number of outpatients and inpatients per day are shown in Table 1.

### 3.2. Water Supply

#### 3.2.1. Types of Water Source

The main water points at the majority (94%) of the health care facilities were improved sources. However, a small proportion (10%) of the level III health centres had unimproved water sources as the main water points. The mean number of years since the main water points were constructed, acquired, or connected was 7 years (sd ± 5.7).

#### 3.2.2. Access to Water Points

Eighty-six percent of the health care facilities had the main water source located on the premises (in buildings or compound). The types and mean number of water points at the different levels of health facility and the proportion of those that were functional are shown in Table 2.

All the water points at the level IV HCs were functional. Overall alternative options for water storage when water is not available at the water points were not available at 44% of the health care facilities, which were mainly the level III HCs (48%).

The mean distance from the inpatient wards to main water points at the health facilities varied from 6 to 16 meters. The mean distance was longest (16 meters) at the level IV HCs. The mean time taken to fill a 20-liter water container varied from 4 to 6 minutes, while the mean queuing time at the water points was 6–8 minutes.

#### 3.2.3. Maintenance of Water Sources

Twelve percent of the health care facilities reported having no funds for maintaining water services. All the facilities with no funds for maintaining water services were the level III HCs. The majority (82%) of health care facilities reported having a WASH risk assessment and maintenance plan for their water services. Twelve percent and 23% of the level IV and level III HCs, respectively, did not have WASH risk assessment and maintenance plans for the water services. The facilities with plans for water services were likely to have funds for maintenance (*p*<0.001).

### 3.3. Sanitation

#### 3.3.1. Availability of Sanitation Services

Improved toilets were available at 96% of the health care facilities visited. The mean numbers of years since the main type of toilet facilities was constructed varied from 8 to 17 years for different levels of health facility. The hospital toilet facilities were the oldest with a mean of 17 years. The number of toilets available at the health care facilities visited varied from 1 to 6. The type of toilet facility, mean number of toilets available by the level of health care facility, and toilet to patient ratio are shown in Table 3.

The hospitals had the poorest toilet to patient ratio (1 toilet for 63 patients) while that of the level IV and level III HCs was similar (1 : 30 and 1 : 29, resp.).

#### 3.3.2. Physical Access to Toilets

Most of the health care facilities (90%) had the toilets separate from the health facility buildings. The mean distance from the outpatient departments to the toilet facilities varied from 30 to 35 meters for different levels of health care facility, while the mean distance from the inpatient wards to the toilet facilities varied from 21 to 22 meters. Fifty percent of the health care facilities had separate toilets for males and females, while only 18% of the health care facilities had toilets constructed to accommodate people with disabilities.

#### 3.3.3. Sanitary Conditions of Toilets

The sanitary conditions of the toilets are shown in Table 4.

Only 38% of the health care facilities visited had the floor of the toilets clean (absence of litter, urine, or fecal matter). The biggest proportion of unclean toilets (71%) was found at the level IV health centres. The majority of health facilities (98%) lacked cleaning materials in the toilets. The frequency with which the toilets are cleaned at most of the health care facilities (66%) was every other day. The toilets at the majority of health facilities (74%) were cleaned by hired cleaners. However, at 6% of the health care facilities, the toilets are cleaned by patient caregivers. At the majority of the care health care facilities (86%), the toilets could close and lock. The capability to close and lock the toilets was significantly associated with cleanliness of the toilet floor (*p* value = 0.037). The majority of health facilities did not have lighting around the toilets at night. Presence of lighting at night around the toilets was associated with cleanliness of the toilet floor (*p* value = 0.023). Pit latrine toilets at 50% of the health care facilities were half full of fecal material, while at 12% of the health care facilities the toilets were full. Sixteen percent of the health care facilities had no mechanism to empty toilets when full or provide alternative options.

### 3.4. Hygiene

The availability of hand washing facilities at the health care facilities is shown in Table 4.

Only 38% of the health care facilities had hand washing facilities at the toilets. The lack of hand washing facilities was most prominent at the level IV health centre toilets (71%). Hand washing facilities were available at other points in most (76%) of the health care facilities. However, both water and soap were present at water points in 24% of the health care facilities. The rest of the points had only water at the time of the survey.

## 4. Discussion

The findings which were observed reveal that main water points at the majority of health care facilities are improved water sources while most have improved sanitation facilities. Availability of hygiene facilities (hand washing amenities and messages) remains very limited in the health care facilities. WASH improvements at government-owned health facilities should take into consideration water supply and sanitation technology improvement and focus on addressing gaps in hygiene facility availability.

In contrast to findings elsewhere [2, 8], improved water source coverage at the health facilities was high, although it remains below the WHO's proposed target of 100% coverage by 2030. The gap in improved water source coverage is mainly at the lower level health facilities (level III HCs). A number of the level III HCs reported not having WASH risk assessment and maintenance plans, which was shown to be associated with availability of funds for water services. It has been suggested elsewhere that service improvements would benefit from comprehensive, facility-based risk assessments, and associated risk management plans [13]. This suggests that capacity to develop risk assessment and maintenance plans for health facility water services impacts directly on service availability and should therefore be addressed at this level.

Although the observed coverage of improved sanitation services is high at the health care facilities, the toilet to patient ratio remains below the WHO recommended standards [12]. The hospitals have the poorest toilet to patient ratio that is about three times below the level recommended. This can be attributed to the high client load and the preferences for high cost flush or pour toilet technology. To improve the toilet to patient ratios, high volume health facilities should consider the construction of cheaper technologies for improved sanitation services to compliment the more costly flush/pour systems, if the SDG target 3.8 is to be met.

The sanitary condition of the available toilets at the health facilities is low and is related to the capability to close and lock the toilet and the availability of lighting in the toilet area. A toilet that can close and lock ensures that the user has adequate time and privacy to properly dispose of fecal matter in a serene manner without undue fear of interruption. Studies conducted elsewhere in Uganda have also found that inpatients and attendants suffered from the dirtiness of the sanitary facilities [24]. To improve sanitary conditions of toilets, emphasis should not be restricted to regular cleaning but also consideration of reinforcing the privacy of and visibility in the toilets.

Consistent with studies elsewhere [2, 7], hygiene standards observed at health care facilities are low and manifested through a lack of the needed amenities and deficiencies in behavior change communication modalities. Technology to improve hygiene standards is the least expensive among all the components of WASH, but this remains the service area with the biggest gap [2]. This indicates a need for interventions to improve attitude and behavior change among both health care providers initially and subsequently the health facility clients.

### 4.1. Limitations of the Study

Although the study was confined to fifty of two hundred eighty-two health facilities in the study area, they represent typical health facilities in a rural setting in terms of client load, infrastructure, and human and financial resources. Therefore, these findings are likely to be similar across regions. The study was confined to primarily government-owned facilities based on the assumption that private health care facilities of a similar level in Uganda are expected to have much higher service standards. This suggests that the finding may not be applicable to contexts that are served by private health care facilities. The data collected from the health facility managers were based on self-reports that are likely to be subject to social desirability bias. As a result, there is a limit to which such responses can be considered accurate by foreknowledge of what, in the view of the respondent, would be a suitable response. The data on hygiene are not reported according to the recommended WHO service levels [23] because the study was conducted (September to November 2015) before these were available. Nonetheless, the findings do carry implications for health service managers, decision-makers, and health care providers in their consideration of the designing and implementing WASH services.

## 5. Conclusions

These findings demonstrate critical gaps in the provision of WASH in health care facilities that need to be addressed to ensure full realization of the Sustainable Development Goals, particularly targets on universal health coverage and access to water and sanitation for all. In order to minimize the risk of health-care-acquired infections, efforts to improve WASH should give prominence to hygiene service interventions in the health care facilities.

Priority should be given to the sustainable provision of hygiene amenities such as soap for hand washing particularly in the high patient volume health care facilities, in this case the level IV health centres and hospitals. This should be complemented by ensuring the availability of educational materials on hand washing such as posters, stickers, and signs at critical positions in the health care facilities.

The poor toilet to patient/caregiver ratios particularly in the high volume health care facilities calls for the provision of cheaper options for improved sanitation in these settings. Overall, availability of services can be improved by institutionalization of WASH risk assessment and maintenance plans that were shown to be linked to availability of funds for maintenance.

## Figures and Tables

**Table 1:** Level of facility by the mean number of staff, outpatients, and inpatients per day.

Facility type (*n*=50)	*n* (%)	Mean number of staff (sd)	Mean number of outpatients/day (sd)	Mean number of inpatients/day (sd)
Hospital (general)	2 (4)	202 (56)	181 (27)	72 (40)
Health centre (level IV)	17 (34)	31 (11)	84 (39)	7 (5)
Health centre (level III)	31 (62)	13 (6)	56 (26)	2 (3)

sd: standard deviation.

**Table 2:** Level of facility by the type of water point, mean number of water points, and presence of alternative options for water storage.

Facility type	Type of water points, *n* (%)		Mean number of water points	Had alternative options for water storage
*n*=50	Unimproved	Improved	*n* (% functional)	*n* (%)
Hospital (general)	0 (0)	2 (100)	47 (89)	1 (50)
Health centre (level IV)	0 (0)	17 (100)	7 (100)	11 (65)
Health centre (level III)	3 (10)	28 (90)	6 (83)	16 (52)

**Table 3:** Level of facility by the mean number of toilets available and stance to outpatient and inpatient population ratio.

Facility type	Type of excreta disposal facility		Mean number of toilet stances	Stance : mean daily OPD and inpatient population ratio
*n*=50	Unimproved, *n* (%)	Improved, *n* (%)	*n*	Ratio
Hospital (general)	0 (0)	2 (100)	4	1 : 63
Health centre (level IV)	1 (6)	16 (94)	3	1 : 30
Health centre (level III)	1 (3)	30 (97)	2	1 : 29

**Table 4:** Level of facility by conditions of the toilets and availability of hygiene facilities.

Facility type	Hospital (general)	Health centre (level IV)	Health centre (level III)	*p* value
Toilet floor clean, *n* (%)	1 (50)	5 (29)	13 (41)	0.651
Intolerable odors present, *n* (%)	2 (100)	13 (76)	16 (52)	0.125
Cleaning materials available, *n* (%)	0 (0)	0 (0)	1 (3)	0.731
Capability to close and lock the toilets, *n* (%)	1 (50)	15 (88)	27 (90)	0.247
Light present at night in the toilets, *n* (%)	1 (50)	4 (24)	16 (50)	0.487
Hand washing facilities present at the toilets, *n* (%)	1 (50)	6 (35)	13 (43)	0.834
Hand washing facilities available at other points of health facility, *n* (%)	2 (100)	14 (82)	22 (71)	0.487
Hand washing facilities have both water and soap present, *n* (%)	0 (0)	2 (14)	7 (32)	0.348
Hand washing posters present, *n* (%)	0 (0)	2 (12)	3 (10)	0.867
Teaching patients/caregivers on proper ways to wash, *n* (%)	1 (50)	16 (94)	29 (94)	0.082

## Data Availability

All data supporting our findings are contained in the paper. There are no restrictions to data sources; however, details of the full data may be accessed through the corresponding author.

## Abbreviations

DALYs: Disability adjusted life years
HCs: Health centres
JMP: Joint monitoring programme
*p* value: Probability value
sd: Standard deviation
SNOWS: Scientists Networked for Outcomes in Water and Sanitation
UNICEF: United Nations International Children's Emergency Fund
WASH: Water, sanitation, and hygiene
WHO: World Health Organization
VIP: Ventilated improved pit.
